# Efficacy of liver free and Chitosan against *Eimeria tenella* in chickens

**DOI:** 10.1186/s12917-024-04124-6

**Published:** 2024-07-15

**Authors:** Zhang Yu, Abdulaziz Alouffi, Ebtsam Al-Olayan, Gungor Cagdas Dincel, Guillermo Tellez-Isaias, Inkar Castellanos-Huerta, Danielle Graham, Victor M. Petrone-Garcia, Beniamino T. Cenci-Goga, Luca Grispoldi, Luís Madeira de Carvalho, Saeed El-Ashram

**Affiliations:** 1 College of Life Science and Engineering, Foshan University, Foshan, Guangdong province China; 2 King Abdulaziz City for Science and Technology, Riyadh12354, Saudi Arabia; 3 Department of Zoology, College of Science, King Saud University, Riyadh, Saudi Arabia; 4 Eskil Vocational School, Laboratory and Veterinary Science, Aksaray University, Aksaray, Turkey; 5 Department of Poultry Science, University of Arkansas, Fayetteville, AR USA; 6 Departamento de Ciencias Pecuarias, Universidad Nacional Autónoma de México (UNAM), Cuautitlan Izcalli, Coyoacán, México; 7 Food Safety and Inspection, Department of Veterinary Medicine, University of Perugia, Perugia, Italy; 8 Centre for Interdisciplinary Research in Animal Health, Faculty of Veterinary Medicine, CIISA, University of Lisbon, Lisbon, Portugal; 9 Associate Laboratory for Animal and Veterinary Sciences (AL4AnimalS), Lisbon, Portugal; 10 Faculty of Science, Kafrelsheikh University, Kafr El-Sheikh, 33516 Egypt

**Keywords:** Liver free, Chitosan, *Eimeria tenella*, Growth parameter, Cecal histology

## Abstract

*Eimeria* spp. are the pathogen that causes coccidiosis, a significant disease that affects intensively reared livestock, especially poultry. Anticoccidial feed additives, chemicals, and ionophores have routinely been employed to reduce *Eimeria* infections in broiler production. Therefore, the shift to antibiotic-free and organic farming necessitates novel coccidiosis preventive strategies. The present study evaluated the effects of potential feed additives, liver free and chitosan, against *Eimeria tenella* infection in White Leghorn broiler female chickens. One hundred sixty-five 1-day-old White Leghorn broiler female chicks were divided into 11 groups (15 female chicks per group), including the positive control group (G1), the negative control group (G2), a chitosan-treated group (G3), a chitosan-treated-infected group (G4), the liver free-treated group (G5), the liver free-treated-infected group (G6), the liver free-and-chitosan-treated group (G7), the liver free-and-chitosan-infected group (G8), the therapeutic liver free-and-chitosan-treated-infected group (G9), the sulfaquinoxaline-treated group (G10), and the sulfaquinoxaline-treated-infected group (G11). Chitosan was fed to the chicks in G3 and G4 as a preventative measure at a dose of 250 mg/kg. The G5 and G6 groups received 1.5 mg/kg of Liverfree. The G7 and G8 groups received chitosan and Liverfree. The G10 and G11 groups were administered 2 g/L of sulfaquinoxaline. From the moment the chicks arrived at Foshan University (one-day-old chicks) until the completion of the experiment, all medications were given to them as a preventative measure. G8 did; however, receive chitosan and liver free as therapeutic supplements at 7 dpi. The current study showed that the combination of liver free and chitosan can achieve better prophylactic and therapeutic effects than either alone. In *E. tenella* challenged chickens, G8 and G9 chickens showed reduced oocyst shedding and lesion score, improved growth performance (body weight, body weight gain, feed intake, feed conversion ratio, and mortality rate), and cecal histology. The current study demonstrates that combining liver free and chitosan has superior preventive and therapeutic benefits than either alone, and they could also be used as alternative anticoccidial agents.

## Introduction

Coccidiosis in poultry is a severe protozoan disease caused by the genus *Eimeria*, which causes massive economic losses to poultry around the world [1–3]. *E. tenella* has a longlife cycle. It starts with an external stage of unsporulated oocysts discharged in feces, then progresses to sporulation and infection. Sporozoites are released from sporocysts entrapped inside each oocyst during the internal phase and invade intestinal epithelial cells in the internal phase when environmentally resistant oocysts (free-living stage) infect chickens [4]. Sporozoites enter intestinal epithelial cells and multiply asexually three times (1st, 2nd, and 3rd schizogony), creating several merozoites each time. Merozoites of the third generation differentiate into micro- and macro-gametes. Gamogony results in the formation of zygotes. Zygotes develop into non-sporulated oocysts, which are released into the environment. Under proper hygrometric and temperature circumstances, sporogony will develop sporulated oocysts in the environment. The merogony phase is the most harmful for the host, in which consecutive intracellular multiplications cause digestive mucosal lesions. Infection may cause damage to the intestinal tract and cecum, resulting in lower feed conversion and production. Feed additives, live vaccines, chemoprophylaxis effectively prevent coccidiosis. However, drug resistance has emerged because of anticoccidial medications’ widespread use and misuse [5]. Thus, there is a continuing desire for novel products that are both safe and effective. Numerous herbs and their extracts are beneficial to broiler chicks infected with *E. tenella*. *Eimeria*-infected chickens treated with *Musa paradisiaca* L. root extract, *Artemisia annua* extract, herbal compound (leaves of *Azadirachta indica* and *Nicotiana tabacum*, flowers of *Calotropis procera*, and seeds of *Trachyspermum ammi* (L.)) or guar meal showed decreased mortality rates and diarrheal scores, improved lesion score and production performance, and decreased oocyst production [6–8]. When dried *Artemesia annua*, a plant containing plant components, such as flavonoids and saponins, was given in the diet at 5% content for three weeks, it decreased the *E. tenella*-induced cecal lesions [9]. Treatment with *Ulmus macrocarpa* extracts improved lesion scores and survival in *Eimeria*-infected chicks [10]. *Sophora flavescens* extract was the most effective in ameliorating bloody diarrhea, mortality rate, lesion score, reduced body weight gain, and increased oocyst production. *Pulsatilla koreana, Sinomenium acutum, U. macrocarpa*, and *Q. indica* were useful [10]. Saponins are phytochemicals obtained from plants that are beneficial in treating chicken coccidiosis. The inclusion of thymol, carvacrol, and saponins reduces *Eimeria* sporozoite invasion into Madin-Darby Bovine Kidney cells, demonstrating the anticoccidial potential for these compounds [11]. After vaccination, a quillaja and yucca (saponin) combination (QY) product can produce variations in oocyst per gram of feces, but QY-fed coccidia vaccinated broilers gain immunity comparable to controls and show significant performance and mortality improvements [12]. Dietary *Ferulago angulata* hydroalcoholic extract, at high inclusion levels in the broiler diet (400 mg/kg), may benefit growth performance and immunological condition in response to a coccidiosis challenge, similar to a probiotic supplement [13]. Garcinia kola methanol crude extract has anticoccidial action against *E. tenella* oocyst [14]. Aside from anticoccidial activities, many natural compounds can alter host immunity and act as prebiotics. Mutamilselvan et al. [15] examined approximately 68 compounds for their potential to treat coccidiosis, including their mechanisms of action. Recent research has reviewed the anticoccidial potency and other favorable effects of numerous phytochemical-herbal treatments [16]. In prior work, chitosan was found to have anti-inflammatory and protective properties against *E. papillata* infection in mice [17]. However, compared to chitosan-rEm14-3-3 NPs and rEm14-3-3, poly (D, L-lactide-co-glycolide)-rEm14-3-3 NP-vaccinated chicken, there was a drastic decrease in relative body weight gain, lesion score, and oocyst reduction ratio in chicken challenged with *E. maxima* [18]. Natural products and herbal medicines are becoming more popular with the domestic poultry industry as more examples of Chinese herbal medicines and the merits of their anticoccidial activity emerge. However, no studies have examined the effects of chitosan or liver free supplements on *Eimeria* infection in chickens, either alone or in combination. More research is needed to evaluate the impact of supplemental chitosan and liver free in lowering *Eimeria*’s adverse effects. This study examines the effects of chitosan and liver free on oocyst shedding, lesion score, growth performance (feed conversion ratio, feed intake, body weight, body weight gain, and mortality rate), and cecal histology in *E. tenella* challenged chickens.

## Materials and methods

### Ethical approval

Foshan University’s ethics committee adopted and accepted the ethical principles for animal regulations (FOSU-03-2020) in March 2020, as stated in the Regulation of Experimental Animal Management of the People’s Republic of China, No. 2, 1988. The chicks were anesthetized with sodium pentobarbital (30 mg/kg body weight) intravenously within 2 min after being removed from their chicken house at the end of the study (7 dpi and 10 dpi). A manual cervical dislocation was used to euthanize the anesthetized chicks. Every attempt was made to reduce the suffering of birds [19].

### Animals and housing

A commercial hatchery (Xinxing Dahua Nong Poultry and Egg Co., Ltd., China) provided 330 one-day-old White Leghorn broiler female chicks, which were kept in a sterilized poultry house at Foshan University, China. Before the main study (165 chicks), we conducted pilot study (165 chicks) to test different doses and observe their effects.

### *E. tenella* oocyst

The sporulated oocysts of *E. tenella* were provided by China Agricultural University’s College of Veterinary Medicine (Beijing, China). To propagate oocysts, ten 14-day-old coccidiosis-free chicks were infected with 2 × 10^4^ sporulated oocysts of *E. tenella* by oral gavage. The feces of infected chicks were collected 7 days post-infection (dpi), and the oocysts were extracted by floating in a saturated salt solution. The oocysts were sporulated in a 2.5% (w/v) potassium dichromate solution at 28 ^˚^C for three days before being washed three times with normal saline. After assessing the total number of oocysts using the McMaster procedure, the sporulated oocysts were diluted to 5 × 10^5^ oocysts/mL in normal saline [2, 3, 20].

### Natural products and anticoccidial drug

Chitosan and sulfaquinoxaline were purchased from Shanghai Zhanyun Chemical Co., Ltd. (Shanghhai, China) and Hubei Zhongmu Anda Pharmaceutical Co. Ltd., respectively. A commercial liver free was provided by Shandong cdVet Natural Products Co., Ltd. (Shandong, China). Shuganbao (舒肝保), Liver free (liver protector), is a veterinary additive that can protect liver function by reducing harmful intestinal flora and toxin absorption. Its main ingredients are *Yucca schidigera* extract, chicory (*Cichorium intybus* L.) root powder, disodium hydrogen phosphate, calcium carbonate, and silicon dioxide, provided by Shandong Shidevitt Biological Engineering Co., Ltd.

### Experimental design

One hundred and sixty-five 1-day-old White Leghorn broiler female chicks free of coccidiosis were purchased from a commercial hatchery (Xinxing Dahua Nong Poultry and Egg Co., Ltd., China) and reared in poultry houses that have been fumigated with ammonia at Foshan University, China. The chicks were equally divided into 11 groups (with 15 female chicks per group), including the positive control group (G1), the negative control group (G2), the chitosan-treated group (G3), the chitosan-treated-infected group(G4), the liver free-treated group (G5), the liver free-treated-infected group (G6), the liver free- and chitosan-treated group (G7), the liver free- and chitosan-infected group (G8), the therapeutic liver free-and chitosan-treated-infected group (G9), and the sulfaquinoxaline-treated group (G10), and the sulfaquinoxaline-treated-infected group (G11). A preliminary experiment (165 chicks) was carried out to determine which group should be chosen for the liver free and chitosan therapeutic applications. Chitosan was prophylactically given at 250 mg/kg to the chicks in G3 and G4. Liver free was given to G5 and G6 at a 1.5 mg/kg dosage based upon our pilot studies, while G7 and G8 received chitosan and liver free. Sulfaquinoxaline (2 g/L) was given to G10 and G11. All treatments were administered prophylactically to the chicks from the time they arrived at Foshan University (one-day-old chicks) until the end of the experiment. However, at 7 dpi, G8 received chitosan and liver free as therapeutic supplements (Table 1).

**Table 1:** Experimental design to study the protective potential of liver free and chitosan against *E. tenella* infection in chickens

Groups		A basal diet with and without natural product supplementation	Infection dose (14-day-old chicks) Sporulated oocysts	Follow-up and sample collection (at day 7 post-infection/21 days old chicks)	Follow-up and sample collection (at day 10 post-infection/24 days old chicks)
**Negative control group**	**G1**	A basal diet without infection or treatment	**-**	7	3
**Positive control group (infection without treatment)**	**G2**	Infection without treatment	6 × 10^4^	7	3
**Chitosan treated group (Prophylactic treatment)**	**G3**	Chitosan was administered at a dose rate of 250 mg/kg daily starting from 1-day-old-chicks to the end of the experiment	-	7	3
**Chitosan treated- infected group (Prophylactic treatment)**	**G4**	Chitosan was administered at a dose rate of 250 mg/kg daily starting from 1-day-old-chicks to the end of the experiment	6 × 10^4^	7	3
**Liver free treated group (Prophylactic treatment)**	**G5**	Liver free was administered at a dose rate of 1.5 gm/ kg starting from 1-day-old-chicks to the end of the experiment	-	7	3
**Liver free treated-infected group (Prophylactic treatment)**	**G6**	Liver free was administered at a dose rate of 1.5 gm/ kg starting from 1-day-old-chicks to the end of the experiment	6 × 10^4^	7	3
**Liver free and chitosan treated group**	**G7**	Liver free and Chitosan from 1-day-old-chicks to the end of the experiment	-	7	3
**Liver free and chitosan treated-infected group (Prophylactic treatment)**	**G8**	Liver free and Chitosan starting from 1-day-old-chicks to the end of the experiment	6 × 10^4^	7	3
**Liver free and chitosan treated-infected group (Therapeutic treatment)**	**G9**	Starting from the day of infection-Therapeutic treatment starting from 14-day-old-chicks to the end of the experiment	6 × 10^4^	7	3
**Sulfaquinoxaline treated group (Prophylactic treatment)**	**G10**	Sulfaquinoxaline was administered at a dose rate of 2 gm/L starting from 1-day-old-chicks to the end of the experiment	-	7	3
**Sulfaquinoxaline treated-infected group (Prophylactic treatment)**	**G11**	Sulfaquinoxaline was administered at a dose rate of 2 gm/L starting from 1-day-old-chicks to the end of the experiment	6 × 10^4^	7	3

### Oocyst counts

A modified McMaster counting chamber, a microscope, and a standard formula were used to compute OPG counts. The McMaster technique was used to count oocysts in feces [21].

### Lesion score of the cecum

We performed a postmortem examination at 7 dpi. We graded dissected birds on a lesion grading scale based on gross lesion severity in the cecum [22]. Lesions included bleeding, thickening of the cecal wall, and mucoid secretions. The lesion score ranges from 0 to 4: A score of 0 means there is no lesion; 1 means a mild lesion; 2 shows a moderate lesion; 3 shows a severe lesion, and 4 shows a more severe lesion.

### Growth performance parameters

Chicken feed conversion ratio (FCR), feed intake (FI), body weight (BW), and body weight gain (BWG) were all documented [23].

#### Body weight (BW)

Throughout the experiment, we carefully weighed each chick.

#### Body weight gain (BWG)

Experimental animals were weighed at the start of the experiment. All birds’ weights were assessed daily at 7:00 a.m. before feeding, and the BWG for each group was computed as follows:

BWG = Final group weight (g) - Initial weight (g)/ Total number of birds.

#### Feed conversion ratio (FCR)

We monitored the chickens’ feed consumption daily. Feed conversion ratios were calculated to assess the impact of different diets:

FCR = Feed consumed (g)/ Final weight (g) - Initial weight (g).

#### Mortality rate

Throughout the experiment, the deaths of White Leghorn female chickens were reported. Mortality rate = Total number of dead birds/ Initial number of birds×100.

### Histopathological examination

The cecum was preserved in 4% formaldehyde in each group and examined histologically on days 7 and 10. These cecal tissues were dehydrated and embedded in paraffin using various ethanol concentrations. The tissue was cut 5 μm thick and stained with hematoxylin and eosin (H & E). An optical microscope was used to examine each sample.

### Statistical analyses

The normal distribution was carried out using the Shapiro-Wilk and Kolmogorov-Smirnov tests. We compared the statistical significance between the control and experimental groups by one-way ANOVA followed by Tukey’s multiple comparisons test. The statistical difference was at the level of *p* < 0.05.

## Results

### Clinical signs

Clinical indications and symptoms were thoroughly followed throughout the experiment after being exposed to *E. tenella*. The chicks in the control group were healthy and had no bloody feces throughout the trial. Clinical indications of severe coccidiosis included lethargy, listlessness, reduced appetite, ruffled feathers, head fall, inability to stand, and death (G2). The infected-untreated group had the most significant death rate (23.1%). In G4 and G11, the mortality rate was the same (8.3%). In the G8 and G9 groups, chickens showed minor listlessness and bloody diarrhea but no deaths compared to the positive control group (G2) (Table 2).

**Table 2:** Mortality rate in different groups from 0 to 7 days after infection (dpi)

Groups		The initial number of chicks	5 dpi	6 dpi	7 dpi	Total mortality rate
**Negative control group**	**G1**	13				0%
**Positive control group (infection without treatment)**	**G2**	13	3			23.1%
**Chitosan treated group(Prophylactic treatment)**	**G3**	13				0%
**Chitosan treated- infected group(Prophylactic treatment)**	**G4**	12		1		8.3%
**Liver free treated group(Prophylactic treatment)**	**G5**	13				0%
**Liver free treated-infected group (Prophylactic treatment)**	**G6**	13				0%
**Liver free and chitosan treated group**	**G7**	12				0%
**Liver free and chitosan- treated-infected group (Prophylactic treatment)**	**G8**	12				0%
**Liver free and chitosan treated-infected group (Therapeutic treatment)**	**G9**	12				0%
**Sulfaquinoxaline treated group(Prophylactic treatment)**	**G10**	13				0%
**Sulfaquinoxaline treated-infected group(Prophylactic treatment)**	**G11**	12		1		8.3%

Note: Days 0 through 4 had no recorded deaths

### Lesion score

**Fig. 1:**
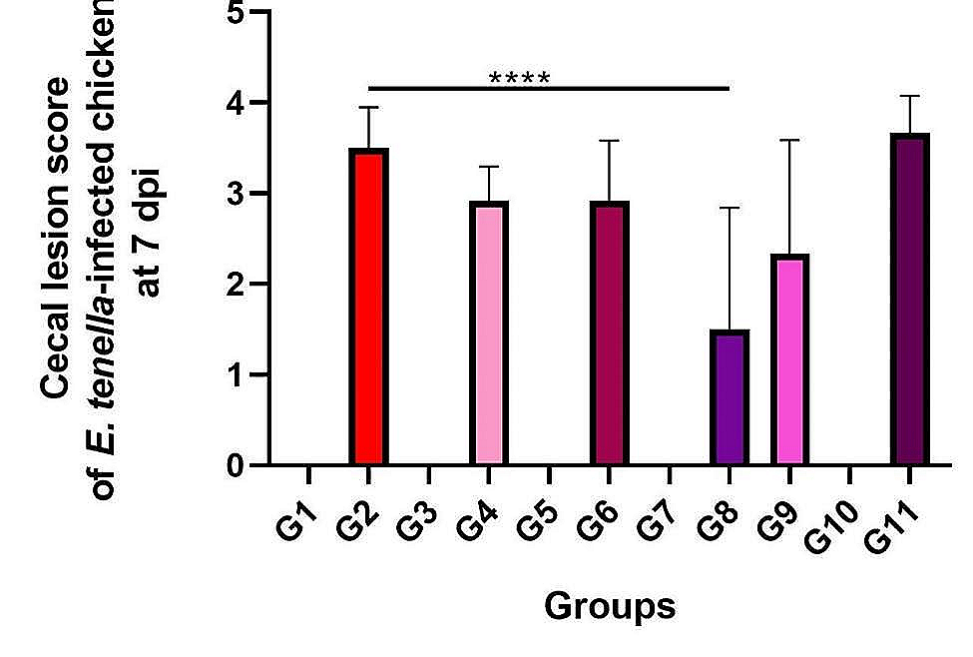
Cecal lesion score of *E. tenella*-infected chickens at 7 dpi

At the end of the investigation, all birds were euthanized by cervical dislocation, and the ceca were promptly inspected to determine the lesion score (Fig. 1). The untreated group (G2) and sulfaquinoxaline-treated infected group (G11) sustained a tremendous amount of cecal damage, while the severity of cecal lesions was reduced in prophylactic and therapeutic groups (G4, G6, G8, and G9). The G8 group exhibited the best anticoccidial effects compared to the other pre-and post-treated groups, and it could significantly minimize the degree of cecal damage (*p* < 0.05).

### Oocyst count

**Fig. 2:**
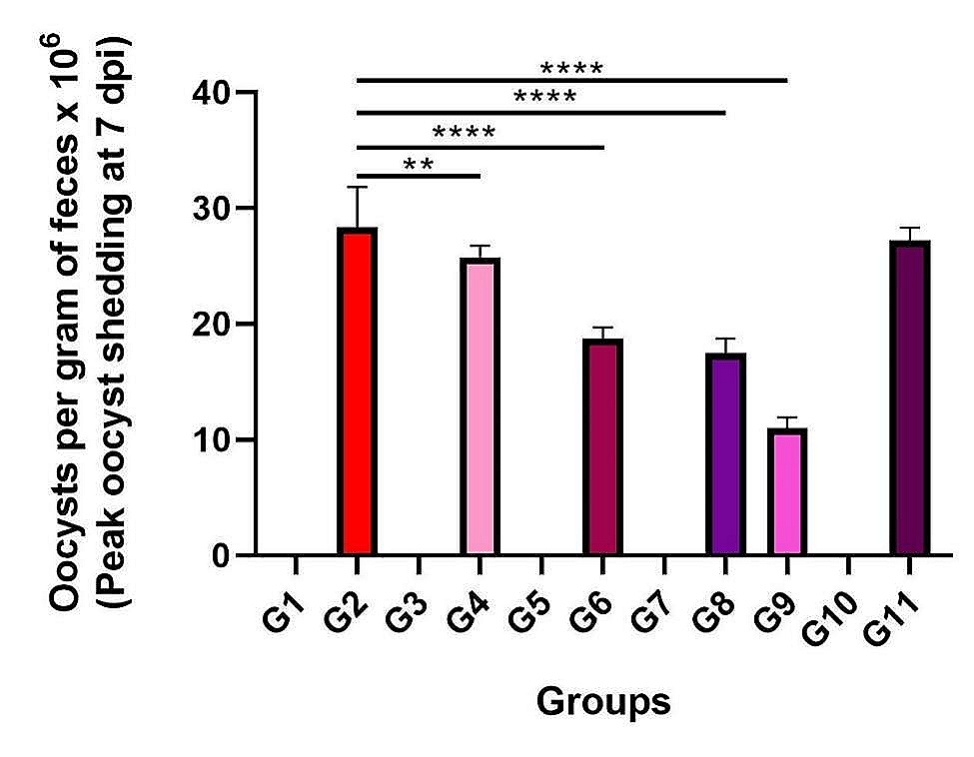
The number of oocysts per gram of feces (×10^6^) at 7 dpi

From day 4 to day 10, feces were collected after birds were infected with *E. tenella*, and the oocyst count was determined (Fig. 2). The peak oocyst shedding day was 7 dpi. In the untreated group (G2) and infected by sulfaquinoxaline (G11) groups, oocyst counts were significantly greater (*p* < 0.05) than those in the other groups. However, there was a substantial difference between the pre- and post-treated groups (G4, G6, G8, and G9) and G2 (*p* < 0.05).

### Growth performance parameters

All growth performance parameters, including BWG and FCR, increased considerably in G8 and G9 compared to G2. However, G6 exhibited the best FCR outcomes in the infected and pre-treated groups.

#### Body weight and body weight gain

**Fig. 3:**
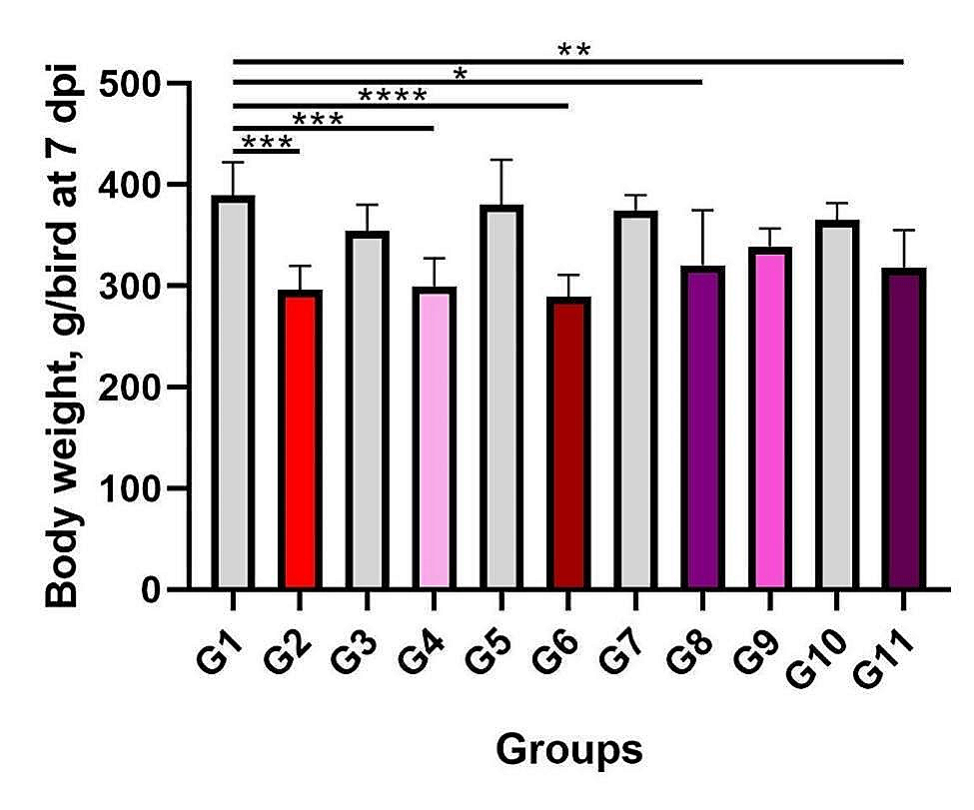
Body weight of chickens (g/bird) at 7 dpi

**Fig. 4:**
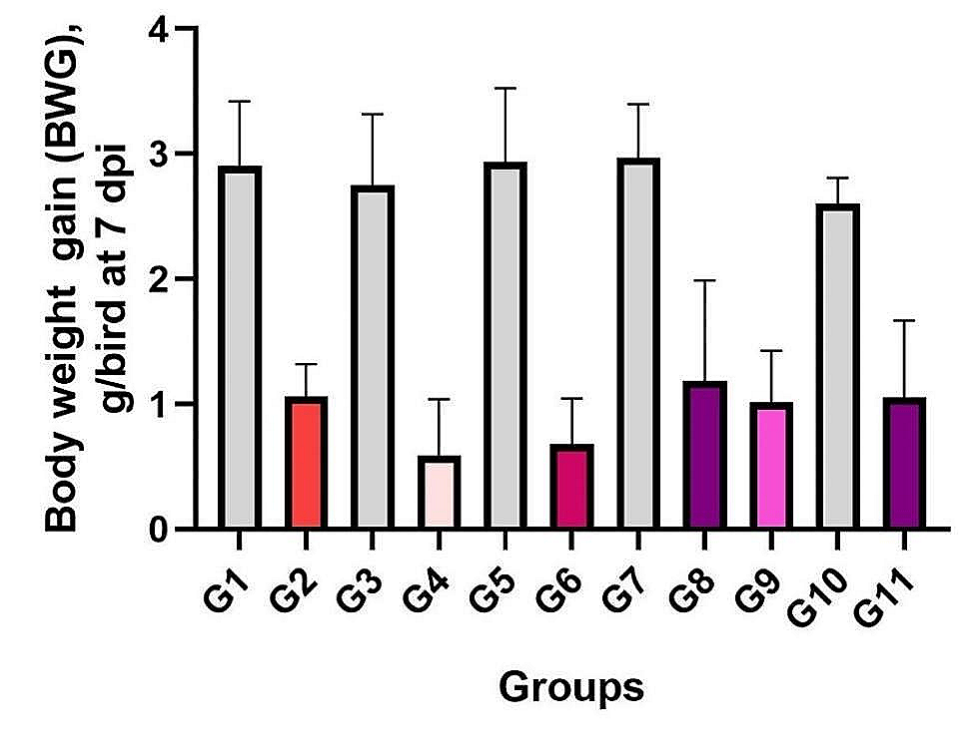
Body weight gain (BWG) of chickens (g/bird) at 7 dpi

G8 and G9 were the superior groups in terms of body weight and body weight gain when compared to G2 and G11 (*p* > 0.05) (Figs. 3 and 4).

#### Feed conversion ratio

**Fig. 5:**
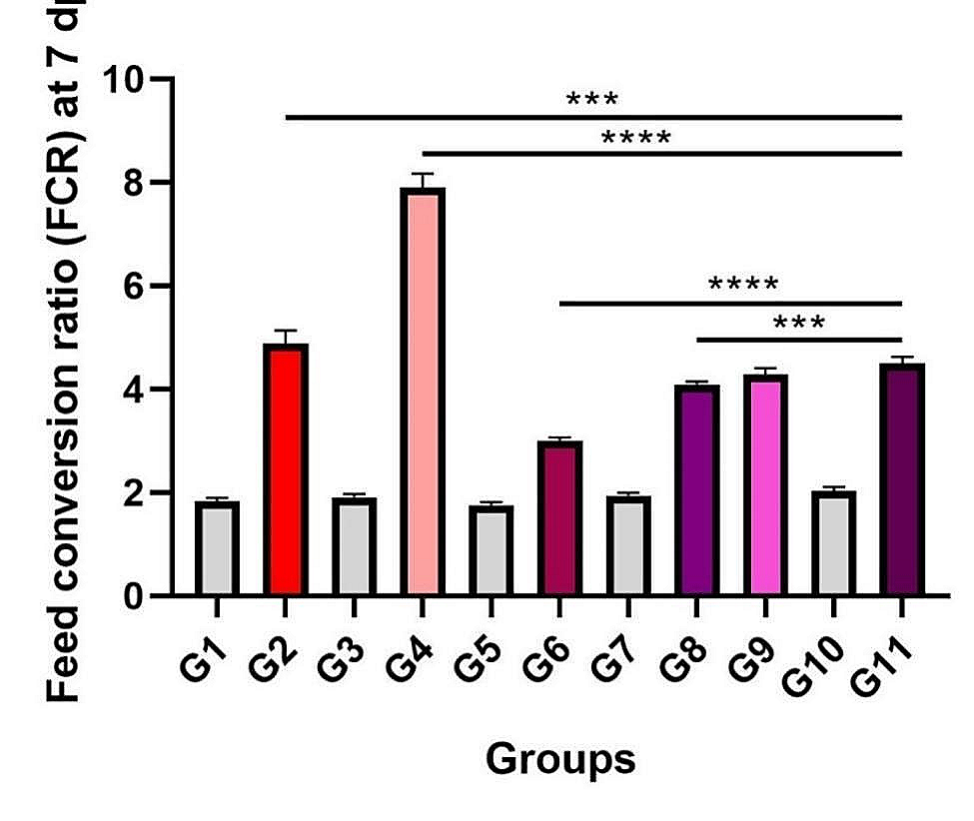
Feed conversion ratio (FCR) chickens (g/bird) at 7 dpi

FCR was reduced in the infected and pretreatment groups by liver free and chitosan supplementation in the G6, G8, and G9 birds. A significant reduction in the FCR of chickens in the G6 and G8 groups was observed as compared to G11 (*p* > 0.05;Fig. 5).

#### Cecal histological examinations

**Fig. 6:**
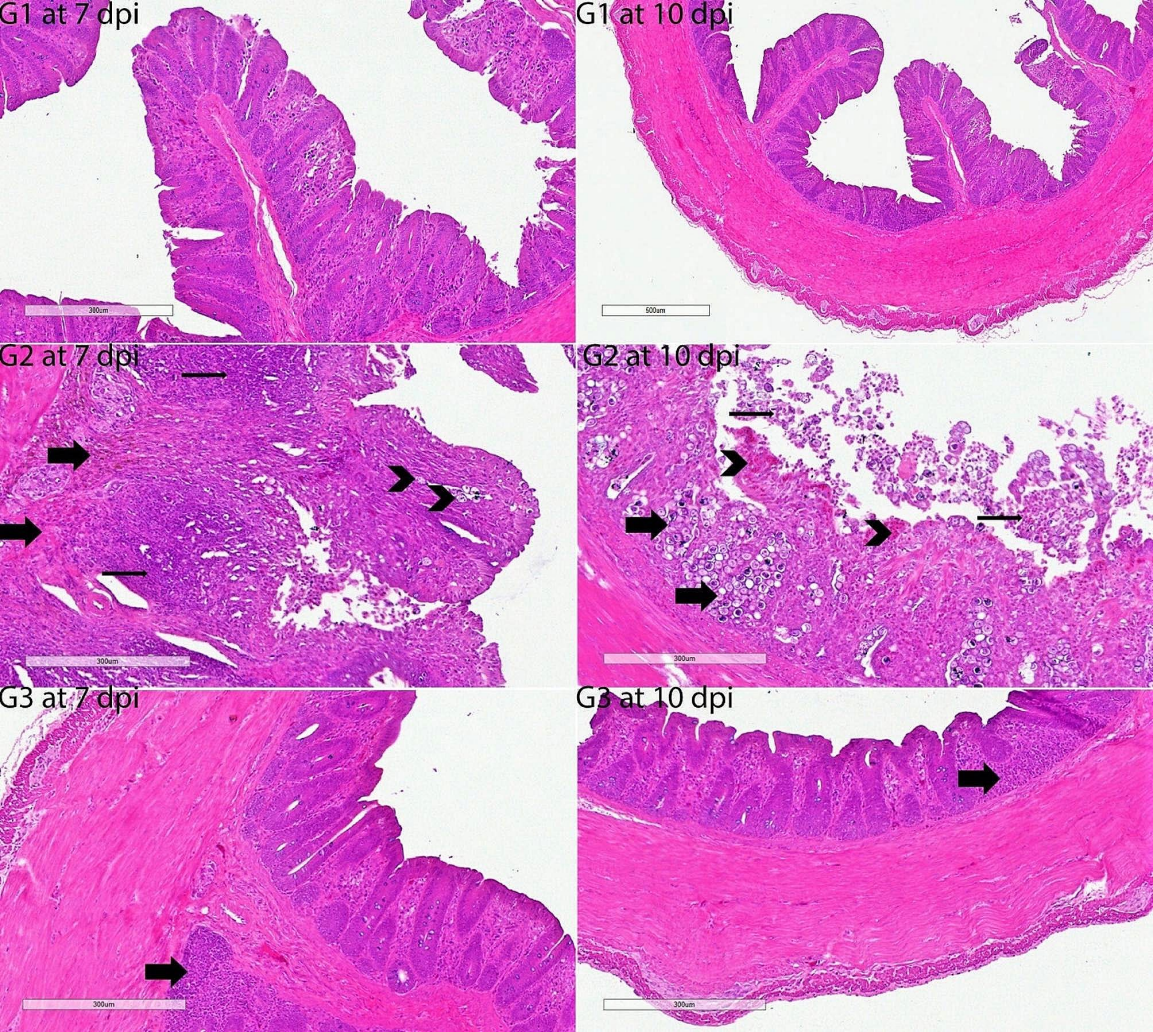
Histopathology of cecal sections of *E. tenella*-infected chickens stained by haematoxylin and eosin (G1-G3 at 7 and 10 dpi). Negative control group (G1) exhibited normal architecture; G1 at 7 dpi, G1 at 10 dpi, and scale bar, 300 μm. A severely detected eosinophilic typhlitis; severe focal cecum hemorrhage and diffuse siderocytes (thick arrows); severe hyperplasia of the GALT and severe inflammatory cell infiltration (thin arrows); common different growth stages (arrowheads) of *E. tenella;* and severe epithelial desquamation accompanied by inflammatory cells, necrotic epithelium, mucus, edema; G1 at 7 dpi; and scale bar, 300 μm. Common different growth stages (thick arrows) of *E. tenella*; marked eosinophil cell infiltration (arrowheads), moderate epithelial desquamation (thin arrows); G2 at 10 dpi; and scale bar, 300 μm. Mild to moderately infiltrative cells in the mucosa/submucosa (thick arrow); G3 at 7 dpi; and scale bar, 300 μm. Mild to moderately infiltrative cells in the mucosa/submucosa (thick arrow); G3 at 10 dpi; and scale bar, 300 μm

**Fig. 7:**
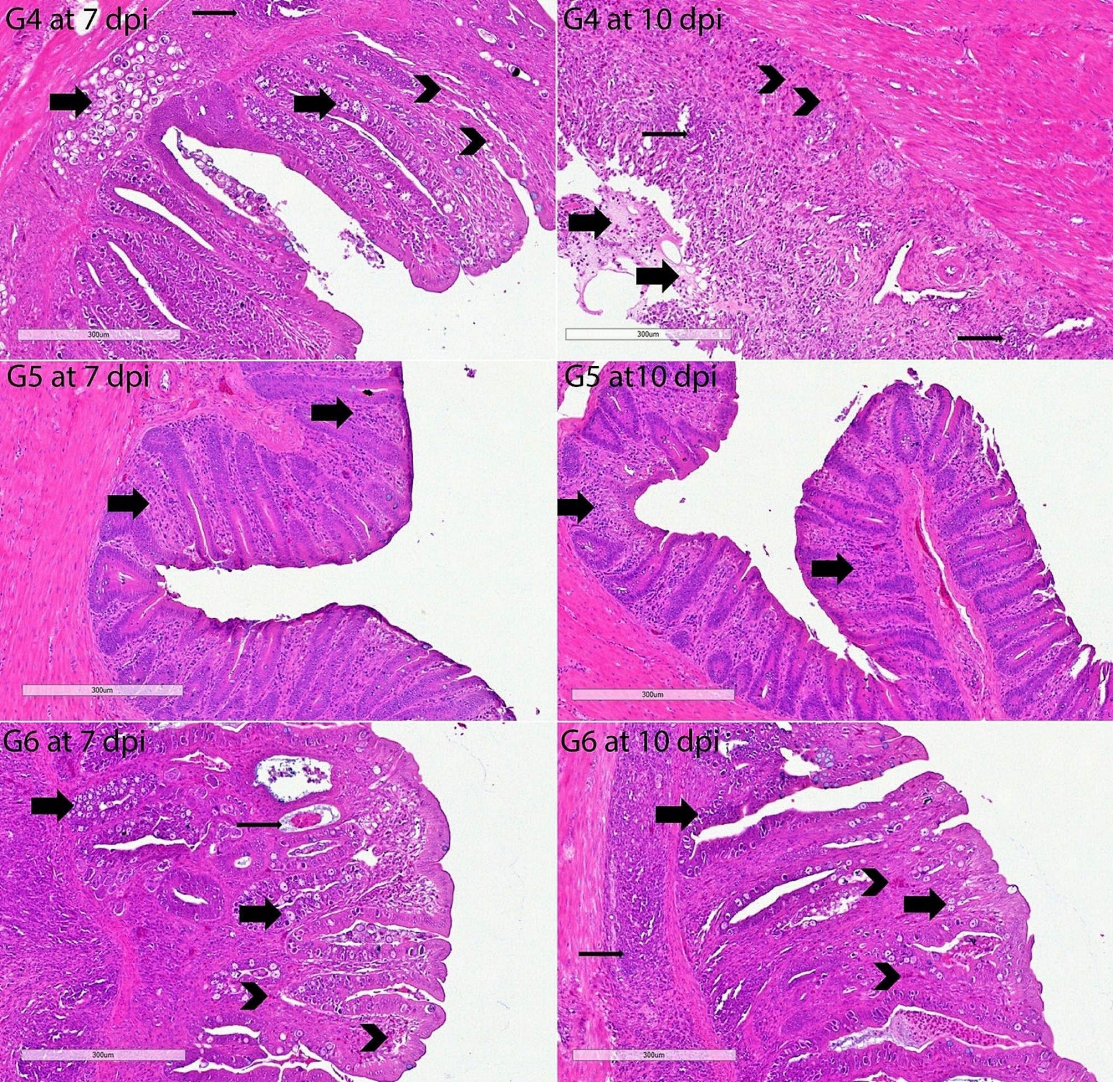
Histopathology of cecal sections of *E. tenella*-infected chickens stained by haematoxylin and eosin (G4-G6 at 7 and 10 dpi). Different growth stages of *E. tenella* in the mucosa and submucosa (thick arrows); diffuse eosinophil infiltrates (arrowheads); moderate hyperplasia of the GALT (thin arrow); G4 at 7 dpi; and scale bar, 300 μm. Greater numbers of eosinophils in the mucosa/submucosa (arrowheads); severe infiltrating immune cells (thin arrow); pseudomembrane (thick arrows) formed by inflammatory cells, necrotic epithelium, mucus, edema; G4 at 10 dpi; and scale bar, 300 μm. Mild cell infiltrations (thick arrows); G5 at 7 dpi; and scale bar, 300 μm. Mild to moderate cell infiltrations (thick arrows); G5 at 10 dpi; and scale bar, 300 μm. Numerous and common different growth stages (thick arrows) of *E. tenella*; slight bleeding (thin arrow) with edema and increased mucus; diffuse eosinophil (arrowheads) and inflammatory cell infiltrates; G6 at 7 dpi; and scale bar, 300 μm. Common different growth stages (thick arrows) of *E. tenella*; diffuse eosinophil (arrowheads) and inflammatory cell infiltrates (thin arrow); G6 at 10 dpi and scale bar, 300 μm

**Fig. 8:**
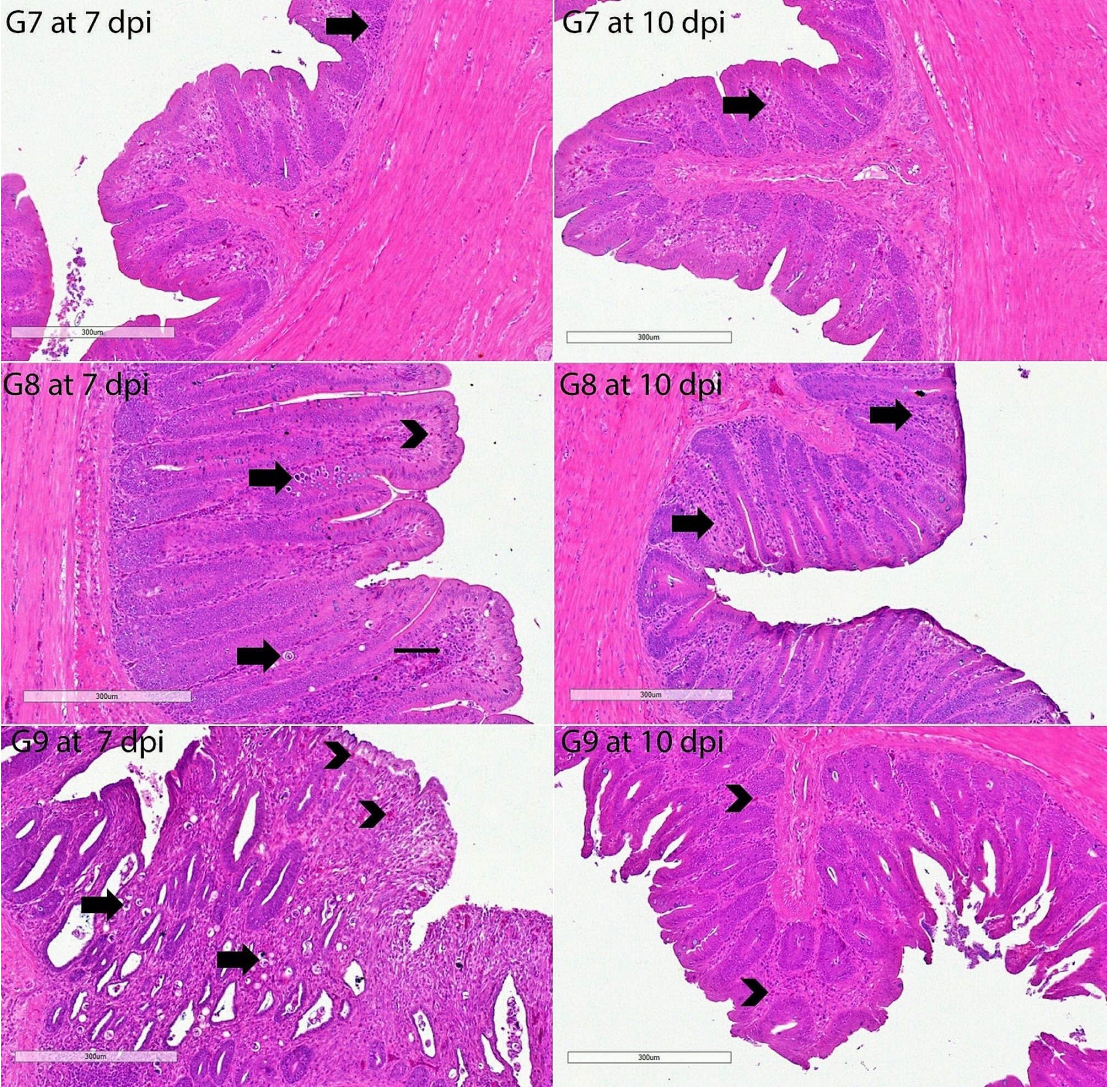
Histopathology of cecal sections of *E. tenella*-infected chickens stained by haematoxylin and eosin (G7-G9 at 7 and 10 dpi). Mild to moderately infiltrative cells in the mucosa/submucosa (thick arrow); G7 at 7 dpi; and scale bar, 300 μm. Mild to moderately infiltrative cells in the mucosa/submucosa (thick arrow); G7 at 10 dpi; and scale bar, 300 μm. A few eosinophil infiltrates (arrowhead); a few different *E. tenella* growth stages (thick arrows); mild immune cell infiltrates (thin arrow); G8 at 7 dpi; and scale bar, 300 m. Very mild eosinophil and inflammatory cell infiltrates (thick arrows); G8 at 10 dpi; and scale bar, 300 μm. Very mild eosinophil and inflammatory cell infiltrates (thick arrows); G8 at 10 dpi; and scale bar, 300 μm. Few different growth stages of *E. tenella* (thick arrows); very mild eosinophil and inflammatory cell infiltrates (thick arrows); G9 at 7 dpi; and scale bar, 300 μm. Very mild eosinophil (arrowhead) and inflammatory cell infiltrates; G9 at 10 dpi; and scale bar, 300 μm

**Fig. 9:**
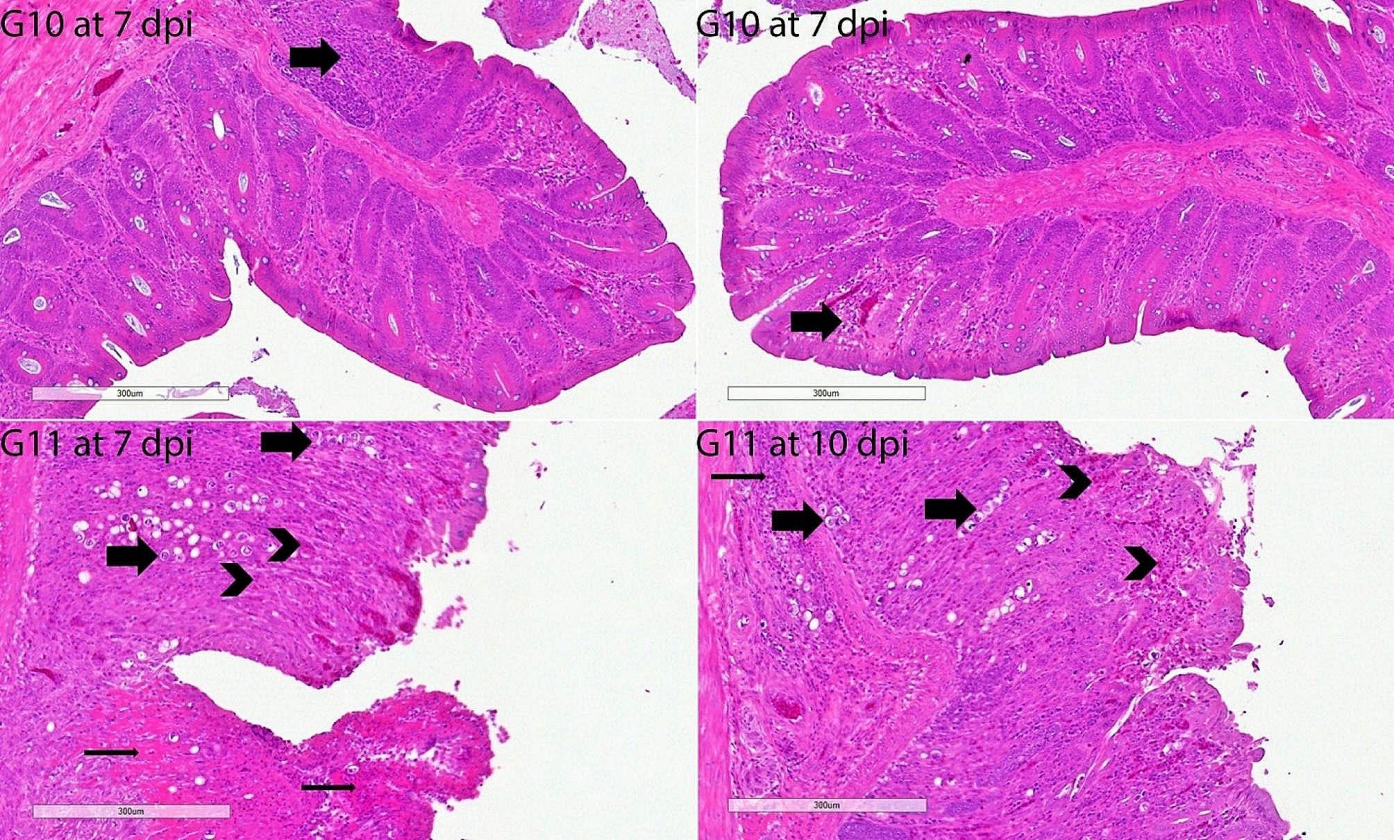
Histopathology of cecal sections of *E. tenella*-infected chickens stained by haematoxylin and eosin (G10-G11 at 7 and 10 dpi). Mild to moderately infiltrative cells in the mucosa/submucosa (thick arrow); G10 at 7 dpi; and scale bar, 300 μm. Mild infiltrative cells in the mucosa/submucosa (thick arrow); G10 at 10 dpi; and scale bar, 300 μm. Severe focal cecum hemorrhage and necosis (thin arrows). Common different growth stages (thick arrows) of *E. tenella*; diffuse eosinophil (arrowheads) and inflammatory cell infiltrates; G11at 7 dpi; and scale bar, 300 μm. Few different growth stages (thick arrows) of *E. tenella*; diffuse eosinophil infiltrates (arrowheads); G11 at 10 dpi; and scale bar, 300 μm

The architecture of H & E-stained intestinal sections from G1 chickens was normal. The G2’s histopathological results were extremely severe. The histopathological results of the untreated positive control group were quite severe. There was enough inflammation to be diagnosed as eosinophilic typhlitis. There was severe focal cecal hemorrhage. Siderocytes were found in abundance in the area of severe mucosal bleeding. There was moderate hyperplasia of gut-associated lymphoid tissue (GALT) in the lamina propria, diffuse necrosis in some areas, and growth stages of *E. tenella*. Severe epithelial desquamation in the cecum was remarkable. Strikingly, the histopathological findings of the G8 and G9 groups were substantially significantly lighter on the 7th and 10th days compared to other infected groups. Some animals showed only a minor infiltration of inflammatory cells and eosinophils. The small number of *E. tenella* endogenous stages in these groups (G8 and G9) was seen as important. Similarly, the groups with fewer histopathological findings than the G11 group were G4 and G6. GALT was shown to have edema, epithelial desquamation, and mild hyperplasia in these two groups (G4 and G6). However, we observed that only one group, G11, did not show a statistically significant difference from the positive control group (G2). In this group (G11), severe mucosal hemorrhages were quite common. Only a few inflammatory cells in the groups didn’t have an infection, like G3, G5, G7, and G10 (Figs. 6, 7, 8, 9).

## Discussion

Coccidiosis is one of the most critical diseases from an economic point of view, but regularly used anticoccidial drugs often result in drug residues in meat and eggs. Natural products can be considered as an effective alternative for anticoccidial treatment in broilers, addressing the emerging resistance of parasites against current treatments. This study introduces two novel natural substances, liver free (employed in the commercial control of mycotoxins) and chitosan, to treat coccidiosis, and their effectiveness outperforms that of sulfaquinoxaline. This study found that chitosan, or liver free, can be used alone or in combination. However, the combined products had a synergistic effect and mitigated the unfavorable impact of chitosan on FCR. Because of its solubility in dilute acids and high water-holding capacity, chitosan forms highly viscous solutions, suggesting greater viscosity in the intestines, which may stimulate intestinal distension in animals and increase satiety. In the current study, this effect might explain why chickens fed chitosan-containing diets had lower feed intake, body weight gain, and higher FCR [24]. Anti-inflammatory and protective effects of chitosan against *E. papillata* infection were observed [17]. However, this contrasts with previous pig studies where no effect of prawn shell chitosan inclusion was seen on the feed conversion ratio [25, 26]. The combined products (liver free and chitosan) administered before and after infection had powerful anticoccidial effects, which might be employed as a prophylactic and therapeutic treatment. The suggested dose of liver free and chitosan is 250 mg/kg and 1.5 g/kg, respectively. Coccidiosis often reduces broiler weight gain because of decreased feed intake, digestibility, and nutrient absorption. Weight gain is generally considered a variable that is more susceptible to coccidiosis and anticoccidial medication. When sporozoites penetrate the epithelial cells of the villi and reach the mucous membrane of the cecum, they cause extensive damage to the epithelium of the cecum, bloody diarrhea, and a significant oocyst discharge [27–31]. In this study, infected chicks became sluggish and depressed, ruffled feathers, and reduced feed intake, likely because of disturbances in intestinal homeostasis, resulting in poor feed intake and metabolism, resulting in reduced body weight gain. All growth performance parameters, including BWG and FCR, increased markedly in the G8 and G9 groups compared to the G2 group. However, G6 showed the best FCR results in the infected and pre-treated groups. The infected-untreated group had the highest mortality rate of all the groups (23.1%). In both G4 and G11, the mortality rate was 8.3%. G6, G8, and G9 all had higher survival rates. In addition, the peak day of oocyst discharge was 7 dpi. Oocyst counts were significantly higher in the untreated group (G2) and the sulfaquinoxaline-treated infected group (G11) than in the other groups (*p* < 0.05); however, the pretreatment and post-treatment groups (G4, G6, G8, and G9) and G2 showed a significant difference (*p* < 0.05). The positive control (G2) and sulfaqinoxaline-infected (G11) groups had the most severe cecal lesions, but the severity of cecal lesions was less in the prophylactic and therapeutic groups (G4, G6, G8, and G9). Compared with other groups before and after treatment, the G8 group showed better anticoccidial effects, and it could significantly reduce damage to the cecum (*p* < 0.05). The main ingredients of the product are *Yucca schidigera* extract, chicory root powder, disodium hydrogen phosphate, calcium carbonate, and silicon dioxide. *Y. schidigera* is the primary ingredient in the commercial product liver free, which is used to ameliorate mycotoxins in the poultry industry. *Y. schidigera* has been found to impact *Eimeria* positively challenged chickens. It was found that adding *Y. schidigera* to the diet of broiler chickens abolished the effect of the *Eimeria* challenge, resulting in comparable or better performance results than the control group [32]. Another study found that dietary supplementation of *Yucca*-derived saponins could ameliorate broiler chickens’ immune and growth responses challenged with *Eimeria* oocysts [33]. The study used a control diet with sham-inoculated birds, a control diet with *Eimeria* oocyst challenge, and two diets with different levels of *Yucca*-derived saponin product and *Eimeria* oocyst challenge. The results showed that the *Yucca*-derived saponin supplementation improved the immune response and growth of the challenged birds. Another study found that both the anticoccidial and the *Y. schidigera* group showed significant improvement in body weight [32]. Overall, the studies suggest that *Y.schidigera* can improve the performance and lower oocyst counts in *Eimeria* challenged chickens. Limited information is available about the effect of chicory (*Cichorium intybus* L.) on *Eimeria-challenged* chickens. However, some studies have explored the potential of chicory in improving the performance of chickens and its antiparasitic activity against various GI parasites in different livestock species [34]. Dietary inclusions of chicory fructans can enhance the recovery of broilers after a challenge with *E. acervulina* and *Clostridium perfringens*, particularly in the pre-inoculation period and the period after the challenge. Chicory can improve chickens’ performance and reduce coccidiosis’s severity. However, more research is needed to understand the impact of chicory on *Eimeria* fully challenged chickens [35]. Limited information about the effect of disodium hydrogen phosphate, calcium carbonate, and silicon dioxide on poultry or *Eimeria* is available. However, some studies have explored the potential of other feed additives in improving the performance of chickens and reducing the severity of coccidiosis. Aluminosilicates, such as zeolite, have been found to increase weight gain, stabilize the gut microbiota, prevent the proliferation of specific intestinal pathogens, and exert an immunostimulatory effect [36]. Chitosan, a natural biopolymer derived from chitin, has been studied for its potential to improve the performance of chickens and reduce the severity of coccidiosis. Synthesized chitosan carriers improved the growth of broiler chicks [37]. The dietary chitosan oligosaccharide could improve growth performance and reduce the severity of coccidiosis in broiler chickens [38]. The combination of liver free and chitosan showed better prophylactic and therapeutic effects than either alone, resulting in reduced oocyst shedding and lesion score, improved growth performance, and cecal histology. Liver free and chitosan were shown to prevent and treat *E. tenella* infection in chickens, making them potential alternatives to conventional anticoccidial agents. Liver free and chitosan could be an improved control measure for *E. tenella* infection, suggesting their possible use in reducing the impact of coccidiosis in poultry farming. These findings were comparable in some ways to the anticoccidial action of *Musa paradisiaca* (L.) root extract, *Artemisia annua* extract, herbal compound (leaves of *Azadirachta indica* and *Nicotiana tabacum*, flowers of *Calotropis procera*, and seeds of *Trachyspermum ammi* (L.) or guar meal that have been reported to decrease the mortality rate and diarrheal score, improve the lesion score and production performance, and reduce the number of oocysts per gram in *Eimeria* challenged chickens [39–42]. *Artemesia annua*, a plant containing flavonoids and saponins, reduced *E. tenella*-induced cecal lesions [9]. Treatment of *Eimeria*-infected chicks with *Ulmus macrocarpa* extract improved survival and lesion scores in *Eimeria*-infected chicks [10]. In terms of bloody diarrhea, survival rate, lesion score, body weight gain, and oocyst discharge, *Sophora flavescens* extract was the most beneficial. *Pulsatilla koreana, Sinomenium acutum, U. macrocarpa*, and *Q. indica* were also beneficial [10]. Because of their diverse components and targets, natural products often show a wide range of pharmacological effects [43]. From a macroscopic perspective, liver free and chitosan might reduce the severity of *E. tenella*-caused cecal lesions. A histological examination revealed that the G8 and G9 groups’ histopathological findings were statistically substantially lighter on the 7th and 10th days than the other infected groups. The minimal number of endogenous *E. tenella* stages in these groups (G8 and G9) was thought to be significant. Similarly, the G4 and G6 groups were shown to have fewer histopathological results than the G11 groups. These findings revealed that liver free, and chitosan inhibited or delayed *E. tenella’*s growth and development.

## Conclusion

The study evaluates the effects of liverfree and chitosan on *E. tenella* infection in chickens, specifically focusing on oocyst shedding, lesion score, growth performance, and cecal histology. The study demonstrates that combining liver free and chitosan has better prophylactic and therapeutic effects against *E. tenella* than using either additive alone. This is evidenced by reduced oocyst shedding and lesion score, improved growth performance, and enhanced cecal histology in *E. tenella*-challenged chickens. The findings suggest that liver free and chitosan could be used as alternative preventive strategies for coccidiosis in poultry, particularly in antibiotic-free and organic farming. The study contributes to the search for novel coccidiosis preventive strategies in the face of the shift towards antibiotic-free and organic farming practices. Further research could focus on investigating the long-term effects of liver free and chitosan supplementation on chickens’ growth performance and health, which could provide valuable insights for their practical application in poultry farming. Comparative studies could be conducted to evaluate the effectiveness of liver free and chitosan in preventing and treating *Eimeria* infections in different poultry species and under various farming conditions. Subsequent research could investigate the effectiveness of the therapies on different *Eimeria* species or combinations of species to acquire full knowledge of their potential as broad-spectrum anticoccidial medicines. Exploring the mechanisms of liver free and chitosan against *E. tenella* could help understand their mode of action and potential synergistic effects. Assessing the economic feasibility and cost-effectiveness of using liver free and chitosan as alternatives to conventional anticoccidial agents in commercial poultry production systems would be beneficial for farmers and industry stakeholders.

## Data Availability

All data generated or analyzed during this study are available from the corresponding author upon reasonable request.
